# A Systematic Review and Meta-Analysis of the Role of Sugar-Free Chewing Gum on Plaque Quantity in the Oral Cavity

**DOI:** 10.3389/froh.2022.845921

**Published:** 2022-03-30

**Authors:** Melanie Nasseripour, Jonathon Timothy Newton, Fiona Warburton, Oluwatunmise Awojobi, Sonya Di Giorgio, Jennifer Elizabeth Gallagher, Avijit Banerjee

**Affiliations:** ^1^Centre for Dental Education, Faculty of Dentistry, Oral and Craniofacial Sciences, King's College London, Guy's Dental Hospital, London, United Kingdom; ^2^Faculty of Dentistry, Oral and Craniofacial Sciences, King's College London, Guy's Dental Hospital, London, United Kingdom

**Keywords:** prevention, polyols, xylitol, children, adults, clinical trials, plaque, sugar-free gum

## Abstract

**Background:**

The aim of this systematic review of published literature was to answer the research question, “What is the difference in the level of plaque quantity, in adults and children who chew sugar-free gum (SFG), compared with those who do not chew SFG, who do not chew gum, or who use alternatives such as probiotics or fluoride varnish?”.

**Methods:**

The systematic review [registered on PROSPERO 2018 (CRD42018094676)] included studies on adults and children with chewing of SFG as the main intervention, where “sugar” referred to monosaccharides and disaccharides. Included studies were in English and corresponded to primary research published between 1946 and 2020. The search conducted spanned all relevant databases using both Medical Subject Headings (MESH) and free text with combinations of “chewing gum,” “sugar-free,” “caries,” “xerostomia,” “periodontal disease.”

**Results:**

Eight articles included plaque quantity as part of their outcomes. Meta-analysis showed that SFG significantly reduced plaque quantity (effect size−0.778; 95% CI−1.167 to−0.39). The correlation between the baseline and the end of study data was assumed to be 0.95 for the control and 0.65 for the SFG group. A sensitivity analysis was conducted with the pre- to post-test correlation, set at 0.95 for the SFG group. This gave an effect size of−1.098 (95% CI−1.539 to−0.656) with *I*^2^ = 89.73%. When looking more specifically at xylitol gum, the results of the meta-analysis showed that it significantly reduced plaque quantity (effect size−0.743; 95% CI−1.148 to−0.338). There was a high degree of heterogeneity between studies with *I*^2^ = 86.0%.

**Conclusion:**

There is some evidence that chewing sugar-free gum, in particular xylitol SFG, reduces the quantity of plaque in the oral cavity in comparison to non SFG chewing or no chewing controls. Further research with improved design, lengthier timeframes and higher number of participants should be considered.

**Systematic Review Registration:**

https://www.crd.york.ac.uk/PROSPERO/display_record.php?RecordID=94676.

## Introduction

The Global Burden of Disease Study 2017 estimates that oral disease affects 3.5 billion people worldwide with oral health-care cost averaging 20% of out-of-pocket health expenditure in most high-income countries [1, 2]. In most low- and middle-income countries, the need for oral health care is beyond the capacity of health-care systems. There is a proven and consistent association between socioeconomic status and the prevalence and severity of oral diseases, which remains across populations in high-, middle- and low-income countries [3]. However, individual risk factors play an important role.

The development of oral diseases such as dental caries and periodontitis is associated with the presence of oral micro-organisms in plaque [2]. It is therefore essential to devise preventive measures which target plaque as a reservoir of micro-organisms specifically or generally. To address the concern mentioned above related to the disproportionate effect of oral disease in the economically and socially-disadvantaged members of communities, a low cost measure such as chewing of sugar-free gum (SFG) should be considered in addition to other well-recognized preventive measures like tooth brushing and flossing [4]. Among reputed international dental associations, The United Kingdom (UK) Oral Health Foundation [5], the European Commission [6, 7], the European Food Safety Authority [7], and the FDI World Dental Federation [8] have recognized the oral benefits of sugar-free gum (SFG) related to stimulating saliva, facilitating natural oral cavity clearance and delivering bacteriostatic ingredients such as xylitol and sorbitol to the oral biofilm [9, 10].

Previous systematic reviews have provided evidence on the efficacy of the use of SFG in reducing dental caries. However, this is only one of several oral diseases and there is a need for research to explore the wider impact of chewing SFG on oral health. Specifically, this manuscript focuses on periodontal disease and among its associated indicators, the quantity of plaque [11, 12].

This paper is part of a wider review which examined the relationship between use of SFG and multiple oral health related outcomes including dental caries; gingival recession; salivary quantity; salivary quality; micro-organism; plaque quantity; plaque quality; periodontal disease; oral disease; and quality of life [13, 14]. This manuscript is presenting specifically plaque quantity (increase or decrease) outcome. The aim is to answer the following research question, “What is the difference in the level of plaque quantity, in adults and children who chew sugar-free gum (SFG), compared with those who do not chew SFG, who do not chew gum, or who use alternatives such as probiotics or fluoride varnish?”.

## Methods

The methodology for this systematic review was registered on PROSPERO 2018 (CRD42018094676) and has been described in a previous publication [11]. A brief summary of the methods used is described below [13].

### Study Design

#### Research Question

The research question (PICO) for this systematic review is:

In adults and children who chew sugar-free gum (SFG), compared with those who do not chew SFG, who do not chew gum or who use alternatives such as probiotics or fluoride varnish, what is the difference in the level of plaque quantity?

#### Outcomes

Multiple oral health related outcomes were examined, grouped into 10 categories: dental caries; gingival recession; salivary quantity; salivary quality; micro-organism; plaque quantity; plaque quality; periodontal disease; oral disease; and quality of life. This review looked specifically at only plaque quantity (increase or decrease). Data were collected on reported adverse consequences (negative effects and harm), alongside acceptability and implementation methods that have been shown to lead to better compliance.

#### Eligibility Criteria

Table 1 details the eligibility criteria for the studies considered in this systematic review.

**Table 1:** Eligibility criteria.

**Inclusion criteria**	**Exclusion criteria**
• Human participants: adults and children • Primary research, published from 1 January 1946 to 31 August 2020 • Study designs: trials including randomized controlled trials (RCTs), crossover trials, pre-post trials, pre-post one arm trials, post-only trials and any design with a comparative arm. Crossover trials were required to have a minimum “washout period” of seven days between intervention arms. • Full text available in English	• Systematic or narrative reviews • Non-experimental studies • Laboratory-based studies • Non-adherence to experimental allocation. That is, any trial where the original participant allocation to intervention / control had been changed on any basis, such as self-reported behavior, assessed level of use of active intervention. • Conference abstracts • Incomplete datasets

#### Interventions

This review only considered studies for inclusion when chewing of SFG was the main intervention. Polyols such as xylitol, sorbitol or malitol satisfied the “sugar-free” criteria as in this review we define monosaccharides (i.e., glucose, fructose, galactose) and disaccharides (i.e., sucrose, lactose, maltose) as “sugars.”

### Search Strategy

**Figure 1:**
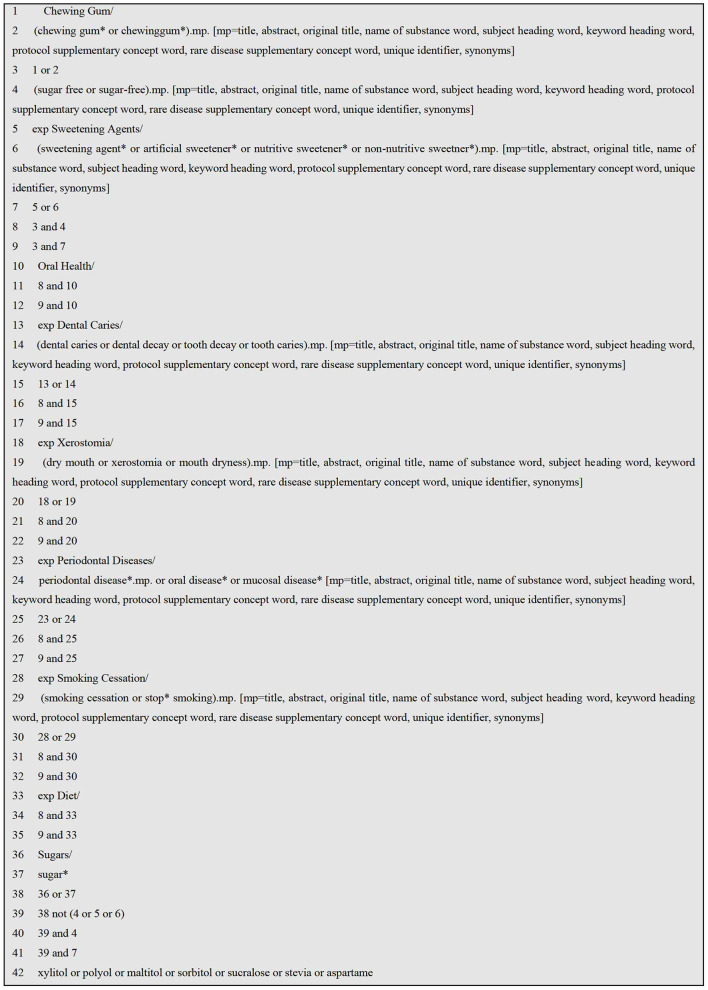
Search Strategy for Ovid Medline, modified for other databases.

The search was designed and conducted by an information specialist (SDG) using both Medical Subject Headings (MESH) and free text with combinations of chewing gum, sugar free, caries, xerostomia, periodontal disease (see Figure 1). The search strategy was applied to one database (OVID Medline) and once confirmed, it was then applied to all other relevant databases with appropriate modifications: Ovid MEDLINE, Ovid EMBASE, Ovid PsycINFO, Scopus, Web of Science, Allied and Complimentary Medicine Database (AMED), Cochrane Central Register of Controlled Trials (CENTRAL), Open Gray, as well as searching Prospero and the Cochrane library of systematic reviews. The search included also reference lists of included studies and any identified relevant systematic reviews.

### Studies Section and Data Extraction

Initial screening of articles identified in the database searches involved independent screening of titles and abstracts by two reviewers (OA / AB), on the basis of the research question (PICO specification) and against the inclusion and exclusion criteria. Following this assessment, the full texts of all potentially relevant studies were checked for eligibility. Disagreements between reviewers was resolved by the input of a third reviewer (JTN).

Data were extracted from each included study based on the pre-determined list of outcomes of interest. This was undertaken in duplicate by three reviewers (OA, MN and JTN) who also developed and piloted the data extraction form prior to extraction. Two reviewers extracted the data from all studies, calling on the third reviewer in the case of disagreement. Differences were resolved through discussion and the input of a fourth reviewer if necessary (AB). Where data were incomplete, study authors were contacted: no response was received from 14, and a further 7 responded but were unable to provide the information requested. Where the same study was reported across several different publications, data were extracted just once but all publications were used to ensure data extraction was maximized across all dimensions under investigation.

All references from identified papers were also reviewed to see if any additional papers met the initial inclusion criteria, as a result of which a seven further papers were included (JTN/MN). After data extraction, several articles were excluded (see Prisma flow chart) for detail of exclusion criteria.

Data on plaque quantity were recorded as well as on the potential effect modifiers such as:

- The intervention: *who delivered it, the setting, details of gum used e.g., ingredients and concentrations, recommended usage e.g., frequency of use, duration of use*.
- Participant characteristics: *age, social class, sample size, diet, pre-existing conditions, risk of population, oral hygiene details*.
- Relevant study details: *number of participants in each arm at baseline and included in analysis, number of withdrawals, follow up period, washout period, unit of randomization, unit of analysis*.
- Bibliographic details: author(s), title, journal, country of origin, year of publication, trial design.

### Data Analysis

Meta-analysis was undertaken using data recorded at baseline and at the end of the study, regardless of when this was. Where there were multiple papers reporting outcomes at successive time points, only the last time point published was included. Where more than one SFG was used, the results were combined, and this was compared to the control group and separate analysis was also undertaken comparing xylitol SFG to a control group. Separate analysis of xylitol-only gums was included since this appeared to be the most frequently adopted SFG in trials and the investigators wished to determine whether any recommendations could be made for xylitol gum specifically. Where the data for either the control or SFG group were available at both baseline and at the end of the study, the paired data were re-created using the method outlined by Borenstein et al. [15].

The correlation between the baseline and the end of study data was assumed to be 0.95 for the control and 0.65 for the SFG group. A sensitivity analysis was conducted with the correlation set at 0.95 for the SFG group.

### Risk of Bias in Individual Studies

Using the Cochrane tool for assessing risk of bias [16], three reviewers (OA, JTN, MN) assessed all included studies independently across six domains: selection, performance, detection, attrition, reporting and “other” biases. The option for disagreements to be resolved through discussion and with the input of a fourth reviewer (AB) as required was available.

### Risk of Bias Across Studies

Whenever concerns were encountered regarding incomplete data, data in graphs or figures, pooled data, incomplete information on key elements of the data extraction form, an attempt was made to contact the authors for clarification. If authors could not be contacted the paper was excluded. If authors responded with clarification or missing data, this information was communicated to the statistician for validity. If valid, the papers were included, and data extraction sheets were completed.

### Summary Measures

The effect size was calculated using the procedure metaeff in Stata v15.1 (StataCorp. 2017. *Stata Statistical Software: Release 15*. College Station, TX: StataCorp LLC). The metaan command in Stata v15.1 was then used to conduct a random effects maximum likelihood meta-analysis and draw forest plots.

### Changes to Protocol Following Commencement of Study

Following the commencement of the study, the decision was made to exclude studies with incomplete outcome data unless contact with the authors could ensure that the data was complete. In the protocol, the analytical strategy stated that analyses would include all covariates (effect modifiers), but these were not included in the analyses reported here. Sensitivity analyses was conducted except for risk of bias which was initially planned but not conducted as there was little variation across the studies for this variable.

## Results

**Figure 2:**
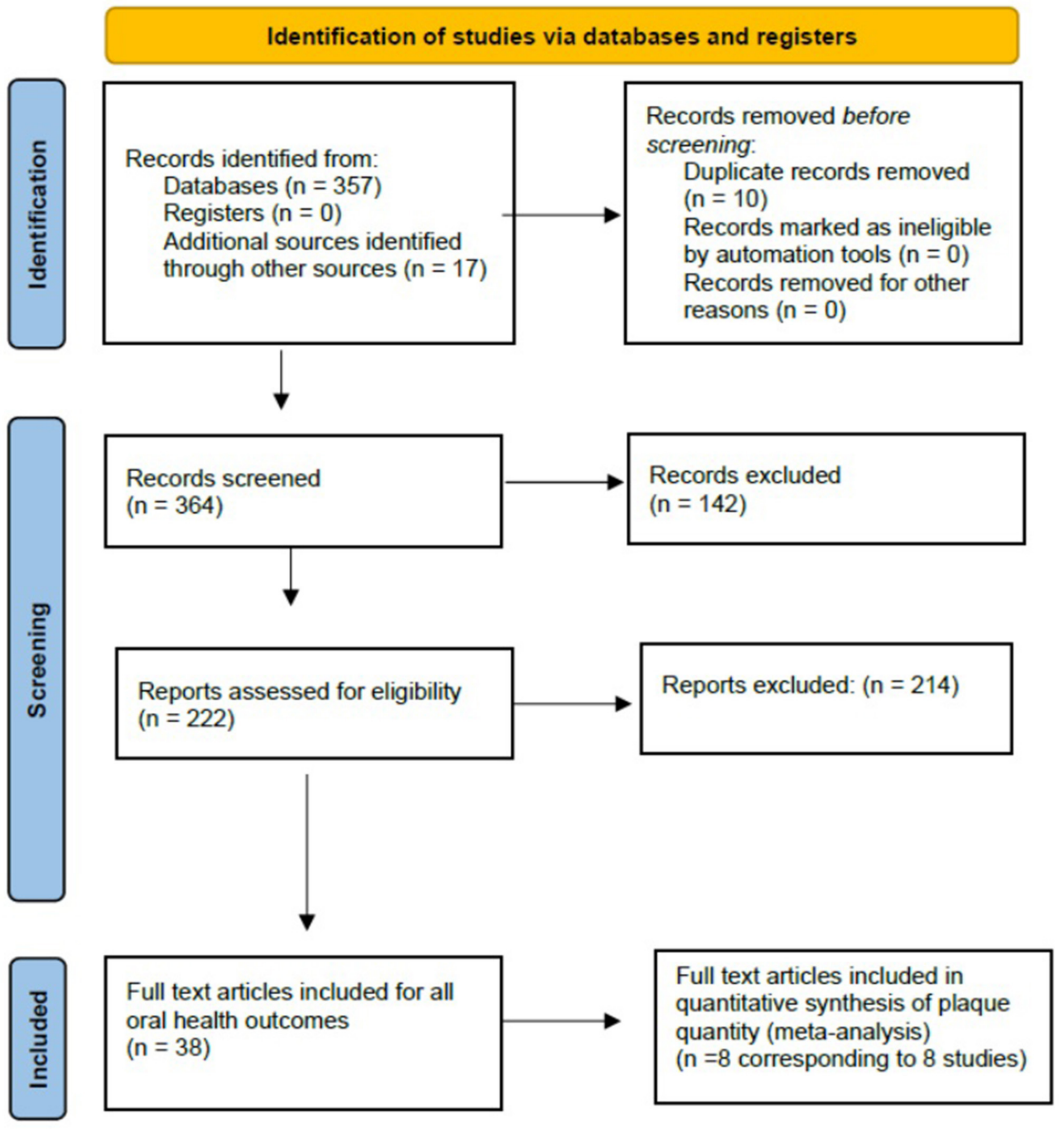
PRISMA flowchart of study identification, screening and inclusion.

The search identified nine papers which included plaque quantity as an outcome, whether by weight or using a plaque index. One study (corresponding to 2 articles) was excluded [17, 18] as the plaque score for the fluoride varnish group (control) at baseline was clearly incorrect and the authors did not respond to requests for clarification. There were therefore eight articles included for analysis. Figure 2 shows the PRISMA flow chart for identification of manuscripts included in this review. Table 2 summarizes the characteristics of the studies included in the review.

**Table 2:** Summary characteristics of included studies.

**Study citation**	**Intervention**	**Participant characteristics**	**Age Range Mean and sd**	**Duration of follow up**	**Study design**	**Control group**	**Intervention arms**	**Outcome measures (count, decline or % change)**
Mouton et al. [19]	xylitol- chewing gum and Sucrose gum	*N* = 96 Adults	Range: 19–25 years Mean and sd unavailable	4 days	RCT	No gum	xylitol- chewing gum and Sucrose gum 6 times daily	Plaque Index (count)
Kandelman and Gagnon [20]	Xylitol gum over 2 years	*N* = 274 children	Range: 8–9 years Mean (sd): Control group: 8.6 ( 0.7) XYL15 group: 8.8 (0.9) XYL65 group: 8.7 (0.8)	2 years	RCT	No gum	Gp1: 15% xylitol gum Gp2: 65% xylitol Three times / day	Plaque index (decline)
Simons et al. [21]	Xylitol gum ACHX: chlorhexidine acetate/xylitol gum	*N* = 111 Adults	Range: 63–99 years Mean and sd unavailable	12 months	RCT	No gum	Xylitol gum 2 times daily ACHX: chlorhexidine acetate/xylitol gum 2 times daily	Plaque index (decline)
Wang et al. [22]	Sugar free gum	*N* = 40 adults	Range: 20 to 39 years Mean 25.8 years sd unavailable	2 weeks	RCT	No gum	Sugar free gum	Plaque quantity (weight) (% change)
Haresaku et al. [23]	xylitol & malatol for 6 months	*N* = 127 adults	Range: 18–53 years Mean 28 years Sd unavailable	6 months	Other – controlled clinical trial?	No gum	xylitol & malatol	Plaque quantity (weight) (decline)
Al-Haboubi et al. [24]	Xylitol gum over 6 months	*N* = 186 adults	Range: over 60 years Mean (sd) 70.2 (7.2)	6 months	RCT	No gum	Xylitol gum over 6 months	Plaque index (decline)
Keukenmeester et al. [25]	xylitol group maltitol group	*N* = 223 Adults	Range: 18–30 years Mean: 21.9 years sd unavailable	21 days	RCT	No gum and no active ingredient gum	Xylitol gum 5 times daily for 21 days Malitol Gum 5 times daily for 21 days	Plaque index (decline)
Saheer et al. [26]	Sorbitol gum Xylitol gum	*N* = 48 young adults	Range: 14–15 years Mean and sd unavailable	14 days	RCT	No gum	Sorbitol gum 2 times daily for 14 days and Xylitol gum 2 times daily for 14 days	Plaque index (decline)

The analysis of the risk of bias within individual studies included in the review is summarized in Table 3. Of the eight studies included in the review, seven were randomized controlled trials (RCTs) and one was not since it took into consideration participant's choice of gum flavor to improve compliance. The population studied was adults, with the exception of one study involving children. The length of the studies was heterogenous, from 4 days to 2 years, as was the chewing protocol. The randomization of participants was unclear for half of the RCTs, high risk for one and low risk for the remaining three. For five of the eight trials, the masking of participants was unclear, the remaining three remained low risk. For two of the trials, a high risk of bias was noted in the reporting of results, and five of the eight were unclear in their outcome reporting.

**Table 3:** Summary of risk of bias of included studies.

**Risk of bias of included studies**	**Study Design**	**Randomization**	**Allocation concealment**	**Masking of participants**	**Masking of outcome assessors**	**Incomplete outcome reporting**	**Selective reporting**	**Other bias**
Mouton et al. [19]	RCT	unclear	high risk	unclear	unclear	low risk	low risk	low risk
Kandelman and Gagnon [20]	RCT	unclear	unclear	unclear	low risk	unclear	unclear	unclear
Simons et al. [21]	RCT	unclear	unclear	unclear	unclear	unclear	unclear	unclear
Wang et al. [22]	RCT	unclear	unclear	low risk	low risk	high risk	unclear	unclear
Haresaku et al. [23]	other	low risk	unclear	unclear	unclear	low risk	unclear	unclear
Al-Haboubi et al. [24]	RCT	high risk	unclear	unclear	low risk	high risk	unclear	unclear
Keukenmeester et al. [25]	RCT	low risk	unclear	low risk	low risk	unclear	unclear	low risk
Saheer et al. [26]	RCT	low risk	low risk	low risk	low risk	unclear	unclear	low risk

**Figure 3:**
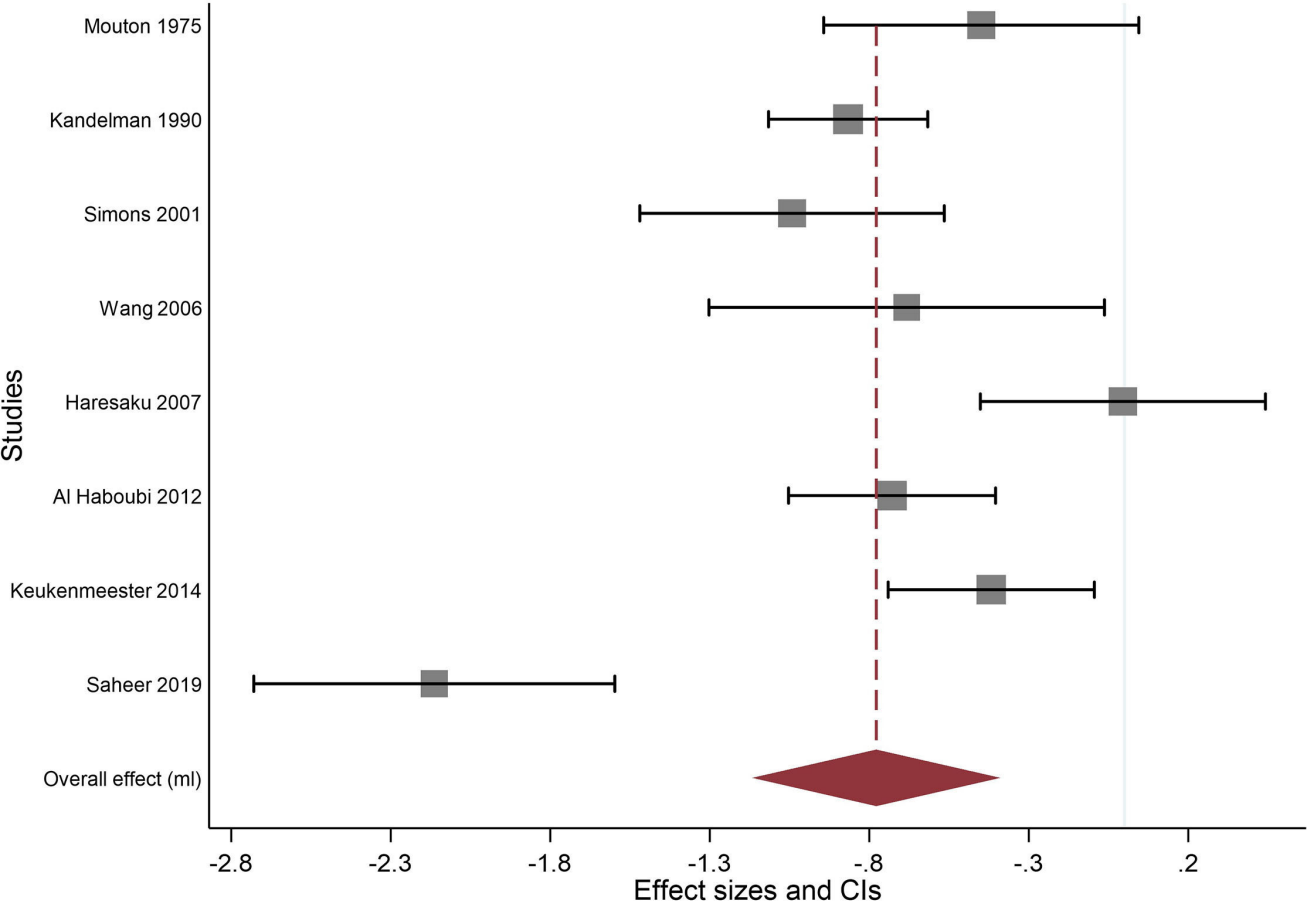
Original weights (squares) displayed. Largest to smallest ratio: 1:30.

The correlation between the baseline and the end of study data was assumed to be 0.95 for the control group and 0.65 for the sugar-free gum group. The results of the meta-analysis can be seen in Figure 3, Table 4 below and show that SFG significantly reduced plaque quantity (effect size−0.778; 95% CI−1.167 to−0.39). There was a high degree of heterogeneity between studies (I^2^ = 86.6%).

**Table 4:** Meta-analysis of any SFG and plaque quantity using the random-effects model by date of publication.

**References**	**Effect [95% Conf. Interval]**			**% Weight**
Mouton et al. [19]	−0.449	−0.943	0.045	12.03
Kandelman and Gagnon [20]	−0.866	−1.116	−0.617	14.07
Simons et al. [21]	−1.042	−1.303	−0.063	10.82
Haresaku et al. [23]	−0.005	−0.452	0.442	12.47
Al Haboubi et al. [24]	−0.729	−1.053	−0.404	13.53
Keukenmeester et al. [25]	−0.418	−0.741	−0.094	13.54
Saheer et al. [26]	−2.164	−2.729	−1.598	11.34
Overall effect (ml)	−0.778	−1.167	−0.390	100.00

A sensitivity analysis was conducted with the correlation set at 0.95 for the SFG group. This gave an effect size of−1.098 (95% CI−1.539 to−0.656) with an I^2^ = 89.73%.

**Figure 4:**
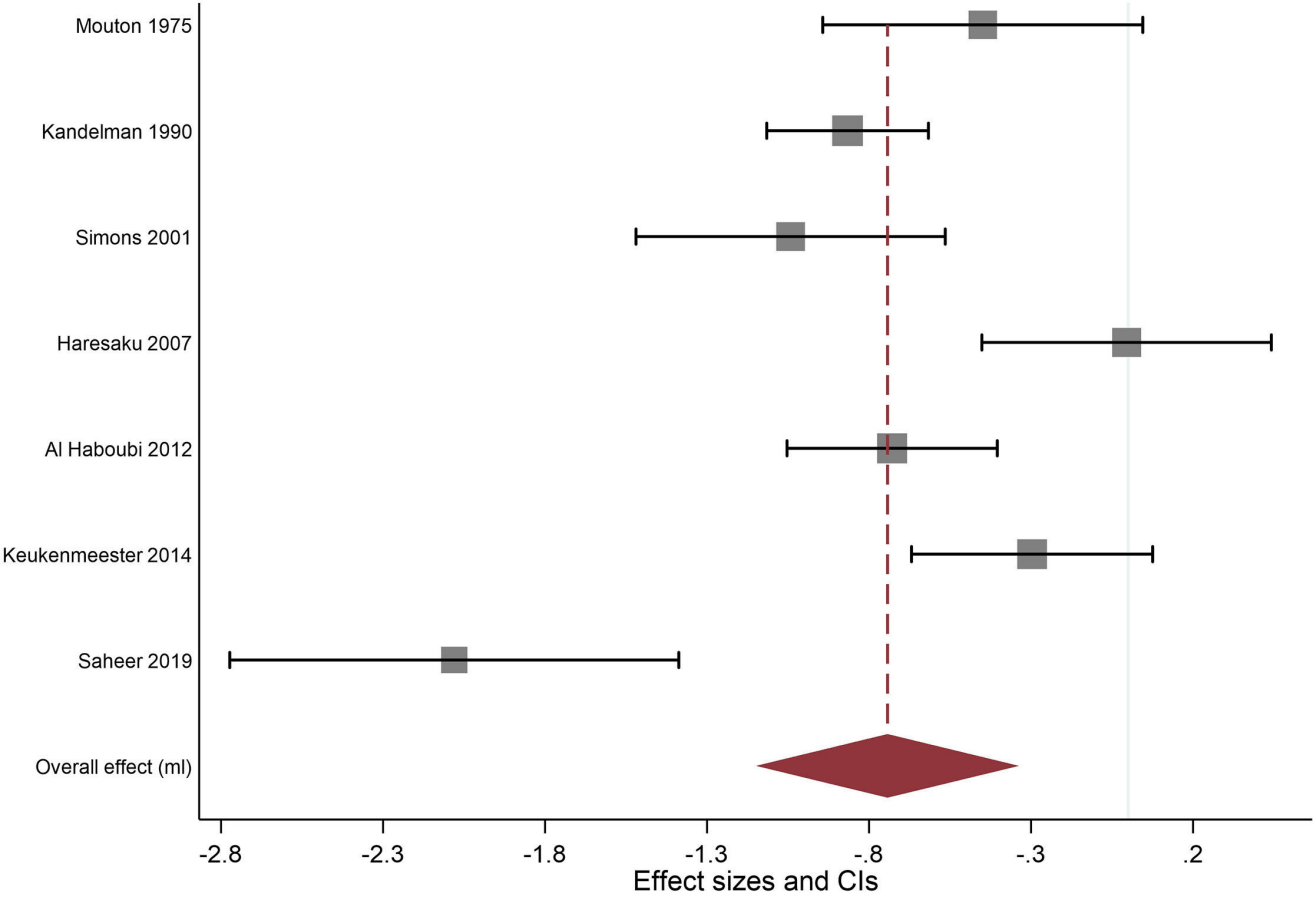
Original weights (squares) displayed. Largest to smallest ratio: 1:41.

A separate meta-analysis of trials where the intervention comprised of xylitol gum only was undertaken and the results can be seen in Figure 4 and Table 5 below showing that xylitol gum significantly reduced plaque quantity (effect size−0.743; 95% CI−1.148 to−0.338). There was a high degree of heterogeneity between studies (I^2^ = 86.0%).

**Table 5:** Meta-analysis of Xylitol SFG and plaque quantity using the random-effects model by date of publication.

**References**	**Effect [95% Conf. Interval]**			**% Weight**
Mouton et al. [19]	−0.449	−0.943	0.045	13.71
Kandelman and Gagnon [20]	−0.866	−1.116	−0.617	16.16
Simons et al. [21]	−1.042	−1.519	−0.565	13.90
Haresaku et al. [23]	−0.005	−0.452	0.442	14.24
Al Haboubi et al. [24]	−0.729	−1.053	−0.404	15.50
Keukenmeester et al. [25]	−0.297	−0.669	0.075	15.03
Saheer et al. [26]	−2.080	−2.773	−1.387	11.45
Overall effect (ml)	−0.743	−1.148	−0.338	100.00

No adverse events were reported in any of the studies.

## Discussion

The oral cavity is home to diverse communities of microbes that live within biofilms and contribute to the formation of dental plaque which is found naturally on teeth. With ecological coexistence between microorganisms and the host, homeostasis and therefore oral health is preserved [27].

An imbalance in this homeostasis, for example in the quantity and quality of dental plaque is implicated in both of the most prevalent biofilm-mediated oral diseases, dental caries and periodontitis [28, 29].

In essence both are mediated by synergistic interactions within bacterial communities and further impacted by specific host-related factors such as diet, behavior and immune system interactions in the case of periodontal disease [29].

Severe periodontal disease, affects almost 10% of the global population. The etiology is related mainly to poor oral hygiene (presence of plaque over time) and tobacco use [3].

The main outcome measurements of oral health have been recognized as presence of plaque, bleeding gums, and gingival inflammation [26]. It is therefore of interest to find adjunctive methods of plaque reduction to standard oral hygiene measures which are often times inadequate in general public but also more specifically in elderly or special care population sub-groups [30].

Looking at the different active agents in the studies presented, it was clear that xylitol-containing SFGs predominated. This meta-analysis of the included studies confirmed that SFG (indiscriminate of the type of sugar-free agent) did significantly reduce plaque quantity. A separate meta-analysis looking at xylitol gum studies alone found that xylitol gum significantly reduced plaque quantity also. In both meta-analyses, a high degree of heterogeneity between studies in study length timeframe, chewing protocol and risk of bias were noted. With the exception of the Kandelman study [20], which focused on children, the findings related to adults.

For Kandelman [20], dental plaque reduction was significant in the chewing-gum groups and up to 21.58% reduction of plaque accumulation was reported in the Wang study [22]. Saheer [26] examined a situation where no mechanical oral hygiene was performed and concluded that the antibacterial properties of SFG in reducing inflammation, as evidenced by the reduction in clinical parameters such as plaque, gingival, and bleeding score, highlighted its potential as an adjunctive oral hygiene measure. Considering the low cost of SFG, it could be a cost-effective measure among oral health high risk target groups. Indeed, the general salivary stimulatory effect from chewing gum could in turn lead to increased clearance in the oral cavity and antibacterial effects via salivary Lysozyme, Lactoferrin, Immunoglobulins, Sialoperoxidase, Cystatins [31]. Simons highlighted the significant positive effect of SFG on plaque and gingival health, despite high plaque indices at baseline [21]. In particular, the antimicrobial agents used (xylitol or chlorhexidine acetate and xylitol) significantly improved periodontal health in the population studied which in this case was older adult occupants of care homes. The Keukenmeister study [25], used a frequently accepted clinical model in the assessment of antimicrobial effects of agents on the development of plaque and gingivitis. The study showed that the groups chewing SFG (xylitol or maltitol) had a significant inhibitory effect on gingivitis scores compared to the gum base control group. For Al-Haboubi [24], as there were no differences in oral hygiene practices from all their participants and no increased salivary flow in the chewing gum group, the improved plaque index may be the result of direct anti-microbial effect from the xylitol on growth and accumulation of the biofilm.

Mouton found that xylitol gum incurred such effects as reduced plaque formation (low weight of plaque, low plaque index), but also diminished the pathogenic qualities of the plaque [19].

Xylitol, as evidenced through microbiological experiments, appears not to be fermented by most micro-organisms present in the oral environment [32–35]. This is of interest when looking at its inhibitory effect on plaque quantity. The Haresaku study [23], highlighted reduction of plaque quantity when chewing xylitol gum for 4–14 days but no statistically significant differences were found at 6 months. The research timeframe was heterogenous for the studies included in the current systematic review spanning from 4 days to 2 years. Only 2 of the 8 studies extended beyond 6 months. Future research should consider setting what might be a realistic and optimal timeframe for the research to explore the clinical effect of regular chewing and explore how it can best be sustained over time.

## Limitations

Several limitations of the current systematic review must be taken into account when considering the conclusions. Non-english literature was not included. Publication bias was not explored and a high heterogeneity in both meta-analyses conducted was found, which may have warranted a sensitivity analysis to identify the variables responsible.

## Conclusions

Within the limitations of this study, the presented systematic review provides some evidence to support the use of sugar-free gum and more particularly xylitol SFG in reducing plaque quantity in adults. Further research should be undertaken to assess the use of SFG and xylitol SFG as an adjunct to established oral health preventive measures to prevent biofilm-mediated diseases including dental caries and periodontitis.

## Data Availability Statement

The raw data supporting the conclusions of this article will be made available by the authors, without reservation.

## Author Contributions

MN contributed to data acquisition, analysis, interpretation, drafted, and critically revised the manuscript. JN contributed to conception, design, data acquisition, analysis, interpretation, and critically revised the manuscript. OA and FW contributed to data acquisition, analysis, and critically revised the manuscript. SD contributed to data acquisition and critically revised the manuscript. JG contributed to design, data analysis, interpretation, and critically revised the manuscript. AB contributed to conception, design, data acquisition, data analysis, interpretation, drafted, and critically revised the manuscript. All authors gave final approval and agree to be accountable for all aspects of the work.

## Funding

This investigator-led independent research financially sponsored by a grant from Mars Wrigley. Sources–The review was funded by Wrigley's. Sponsor–The review's sponsor was King's College London. The funder had no role in the development of this protocol, data analysis or interpretation of results. All members of the review team are employed by the sponsor, King's College London.

## Conflict of Interest

The authors declare that the research was conducted in the absence of any commercial or financial relationships that could be construed as a potential conflict of interest.

## Publisher's Note

All claims expressed in this article are solely those of the authors and do not necessarily represent those of their affiliated organizations, or those of the publisher, the editors and the reviewers. Any product that may be evaluated in this article, or claim that may be made by its manufacturer, is not guaranteed or endorsed by the publisher.

## Abbreviations

SFG: sugar-free gum
WHO: world health organisation
SM: *streptococcus mutans*
UK: united kingdom
EFSA: european food safety authority
FDI: fédération dentaire internationale
ES: standardized effect size
CI: confidence interval
RCT: randomized control trial
MESH: medical subject headings
ovid: object, view and interaction design
EMBASE: excerpta medica dataBASE
MEDLINE: medical literature analysis and retrieval system online
PsycINFO: psychological information database
AMED: allied and complimentary medicine database
CENTRAL: cochrane central register of controlled trials
PICO: population intervention comparison outcome
PF: prevention factor
SMD: standardized mean difference.
